# The Triply Twisted Heart: Cyanosis in an Adult With Situs Inversus, Levocardia, Double Outlet Right Ventricle, and Malposition of the Great Arteries

**DOI:** 10.14740/cr440w

**Published:** 2015-12-16

**Authors:** Jaime Alfonso M. Aherrera, Jose Donato A. Magno, Celia Catherine C. Uy, Lauro L. Abrahan, Helga F. Sta. Maria, Rodel R. Buitizon, Raul D. Jara

**Affiliations:** a Section of Cardiology, Department of Medicine, University of the Philippines, Philippine General Hospital, Philippines; b Department of Cardiology, St. Luke’s Medical Center, Global City, Philippines

**Keywords:** Malposition of the great arteries, Atrioventricular septal defect, Situs inversus

## Abstract

We present a case of a 19-year-old female presenting with cyanosis since birth. The major anomaly demonstrated was a “triply twisted heart” with a balanced physiology, allowing her to survive into adulthood. Non-invasive imaging was done using 2D and real-time 3D (or 4D) echocardiography with multi-slice imaging from 4D volume datasets. Findings were confirmed using cardiac magnetic resonance imaging (MRI). A segmental approach revealed atrial and visceral situs inversus, levocardia, atrioventricular discordance, and ventriculoarterial discordance. Both the aorta and pulmonary artery were malposed and arise from the right ventricle (double outlet right ventricle or DORV). There was also a complete atrioventricular septal defect (CAVSD) associated with a functional single atrium and a functional univentricle (single ventricle). Other findings include a severe pulmonic stenosis (PS), preserved right and left ventricular systolic function, and a normal pulmonary arterial pressure. She also had a persistent left superior vena cava (SVC) that drains into the morphologic right atrium, while the right-sided SVC drains into the morphologic left atrium. A multidisciplinary team deemed that management be palliative. She is on regular follow-up at our clinics for non-invasive monitoring. To our knowledge, this is the first reported case in an adult with this combination of anomalies.

## Introduction

In the 1970s, Richard Van Praagh proposed a “segmental approach” which divided the cardiac structures into three major segments: the atria, ventricles, and the great arteries. Embryologically, the viscera-atrial situs dictates the location of the atria, while the bulboventricular loops dictate the location of the ventricles and great arteries [1]. Robert Anderson had his own version of the segmental approach: atrial arrangement (situs), atrioventricular connections, and ventriculoarterial connections [2]. Evidently, non-invasive modalities play a major role in the diagnosis of congenital heart defects. We present a case of a young adult with a rare combination of multiple congenital cardiac defects resulting in a “triply twisted heart” diagnosed by echocardiography and cardiac magnetic resonance imaging (MRI).

## Case Report

A 19-year-old female sought consult for exertional dyspnea. She was born at full term via spontaneous vaginal delivery to a then 38-year-old G5P4 mother at home assisted by a midwife. At 3 months of age, she was noted to have cyanosis while crying. In the interim, she had easy fatigability, cyanosis on exertion, poor weight gain, and dyspnea. While growing up, back deformity was also noted which was associated with occasional pain. No consults were done due to financial constraints. One month prior to admission, she experienced worsening heart failure symptoms and associated back pain.

Her blood pressure was 100 - 110/60 mm Hg on all extremities with a heart rate of 80 - 90 beats per minute. She had a normal rate and regular rhythm, precordial bulge, a grade 2/6 systolic ejection murmur at the left upper sternal border, a grade 4/6 pansystolic murmur at the left parasternal border from the second to the fourth intercostal space, and a loud P2. Pulses were full and equal, but she had clubbing and an O_2_ saturation of 75-78% on all extremities.

Electrocardiogram showed sinus tachycardia, right axis deviation, and biventricular hypertrophy. A chest radiograph showed a normal sized heart with pleural effusion on the right and bilateral pulmonary nodules. A chest CT scan revealed findings suggestive of extensive pulmonary tuberculosis with endobronchial spread. Incidentally, the liver was seen on the left, while the spleen and stomach were on the right, suggestive of situs inversus but with levocardia. Workup of the spine revealed cervicothoracic levoscoliosis and thoracic dextroscoliosis, with no evidence of Pott’s disease. Sputum AFB smears were all positive. The initial impression was a cyanotic congenital heart disease, probably a tetralogy of Fallot (TOF), situs inversus with levocardia, congenital scoliosis, and pulmonary tuberculosis.

**Figure 1:**
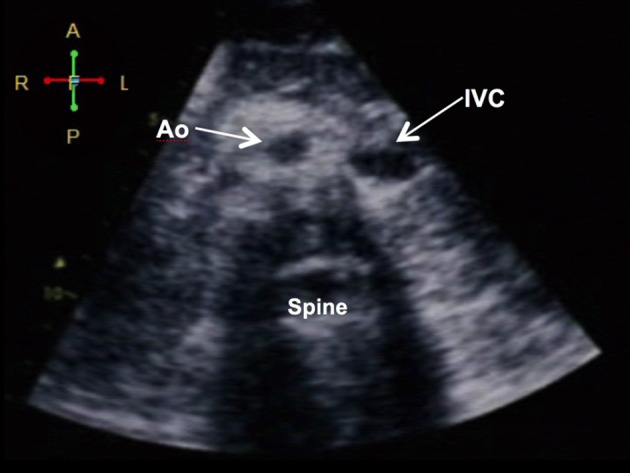
Demonstration of situs inversus. (A) Subcostal view showing the aorta (Ao) and the inferior vena cava (IVC) in relation to the spine: the aorta (more pulsatile) is at the right while the IVC (collapsible) is on the left (visceral situs inversus). The liver is on the left and the spleen and stomach are on the left (not shown).

**Figure 2:**
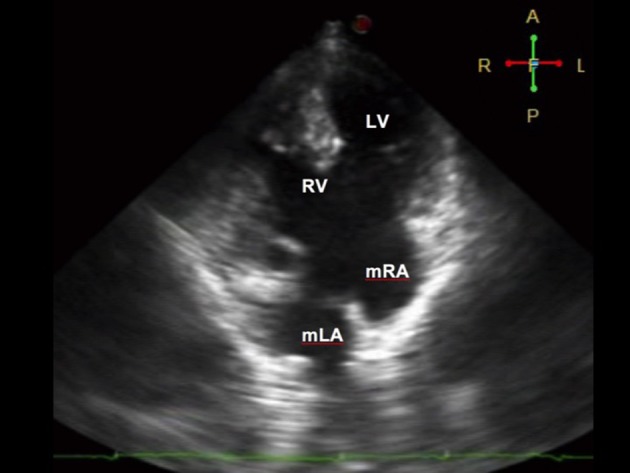
Four-chamber view on 2D echocardiography during diastole showing a complete atrioventricular (AV) septal defect Rastelli type A. There is a common AV valve, which makes it difficult to use the valves to identify the ventricles. Note that the moderator band is seen on the ventricle of the right (morphologic RV). The morphologic right atrium (mRA) drains into the left ventricle, while the morphologic left atrium (mLA) drains into the right ventricle, suggestive of atrioventricular discordance. The RV (systemic chamber) is more hypertrophied than the LV.

**Figure 3:**
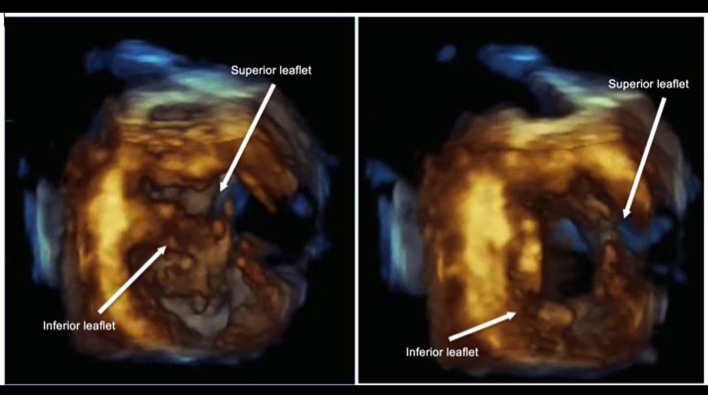
3D echocardiography demonstrating a common AV valve during systole (left) and diastole (right) taken at short axis view with its corresponding M-mode. No significant regurgitation was noted on color flow Doppler.

A segmental echocardiographic approach was done revealing levocardia, atrial and visceral situs inversus, and atrioventricular discordance. The malposed aorta and pulmonary artery both arose from the right ventricle (double outlet right ventricle (DORV) with malposition of the great arteries). There was also a complete atrioventricular septal defect (CAVSD) Rastelli type A with a common (single) atrioventricular valve, visualized in detail on real-time 3D echocardiography. She had a severe pulmonic stenosis (PS), explaining the normal pulmonary arterial pressure. She had preserved systolic function of both RV and LV. Figures 1-3 show representative echocardiographic images.

**Figure 4:**
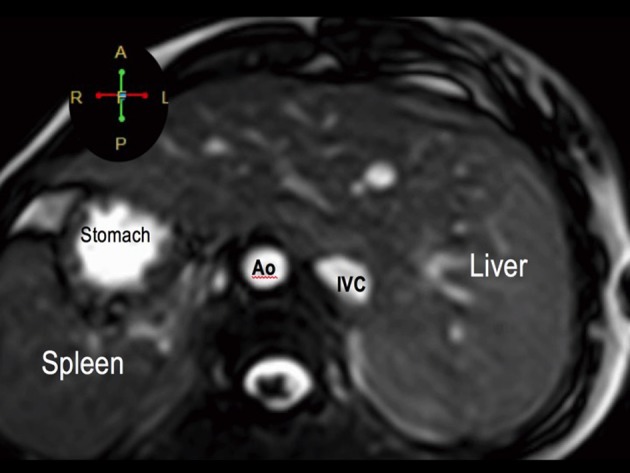
Visceral situs inversus seen on cardiac MRI. Axial cut at the level of the left-sided liver and right-sided spleen and stomach. The aorta (Ao) is on the right while the inferior vena cava (IVC) is on the left.

**Figure 5:**
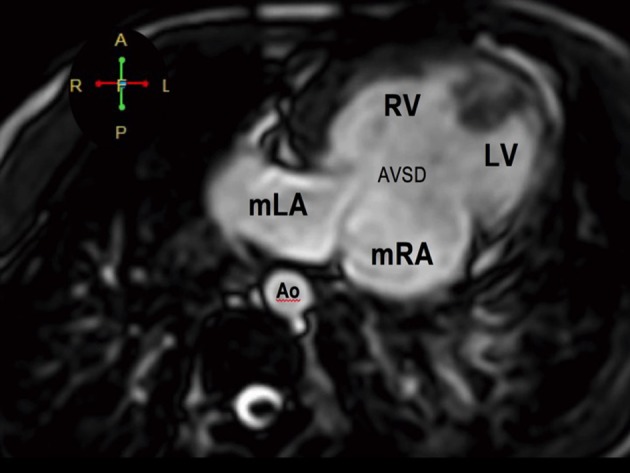
Atrioventricular discordance on cardiac MRI. The morphologic LA drains into the morphologic RV while the morphologic RA drains into the morphologic LV. There is a complete atrioventricular septal defect (AVSD).

**Figure 6:**
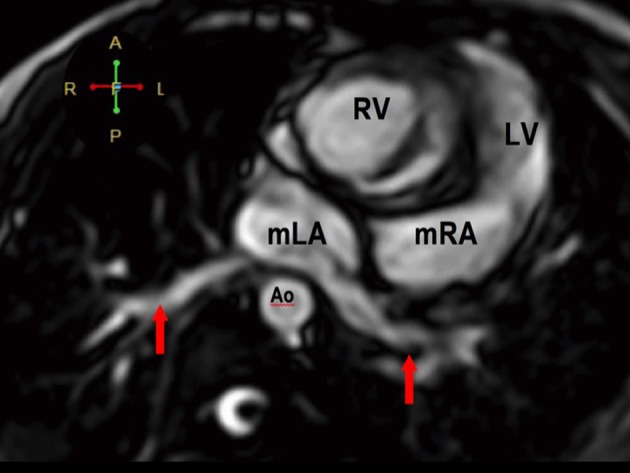
The pulmonary veins drain into the morphologic left atrium located on the right side (atrial situs inversus).

**Figure 7:**
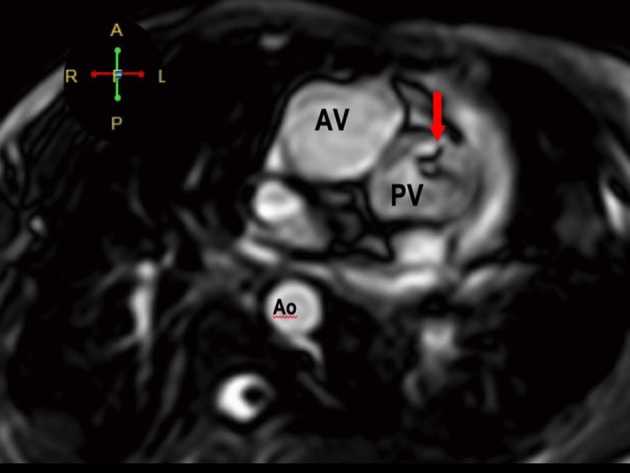
The RV eventually drains into the malposed aorta (Ao) and main pulmonary artery (MPA), indicative of DORV with malposition. There is also note of pulmonic stenosis (arrow).

**Figure 8:**
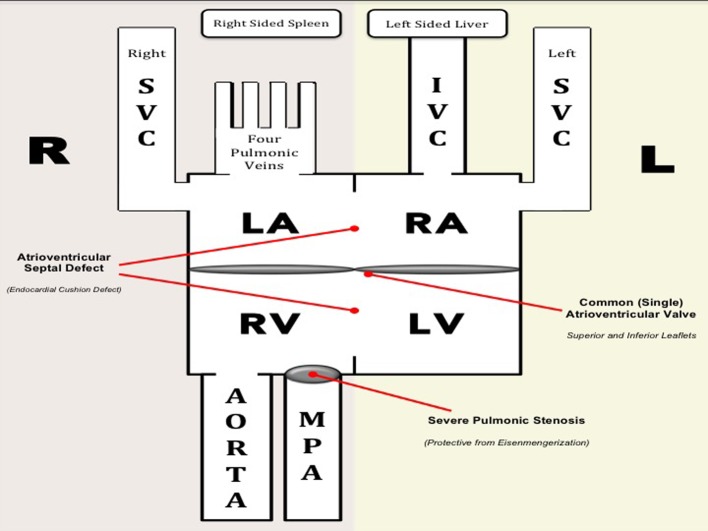
Hemodynamics of our patient. Deoxygenated blood from the IVC and left SVC enter the morphologic RA and blood from the right SVC enter the morphologic LA. Oxygenated blood from the four pulmonary veins drain into the morphologic LA. The morphologic RA and LA are separated by a short segment of the interatrial septum. Functionally, deoxygenated blood from the three vena cavae and oxygenated blood from the pulmonary veins enter a common (single) atrium. Mixed blood from the atria drain across a common AV valve into a functionally univentricle. Anatomically, both ventricles are separated from each other by a short interventricular septum. Mixed blood from the functional univentricle drain into the aorta and main pulmonary artery, both of which arise from the RV (DORV). Note that there is a severe pulmonic stenosis which is protective from the development of pulmonary hypertension (i.e. Eisenmengerization). Diagram on the right represents the normal anatomy.

A cardiac MRI was done to confirm these complex congenital heart defects (Figs. 4-7). Findings were consistent with echocardiography, but with the addition of a persistent left superior vena cava (SVC). Figure 8 gives a diagrammatic representation of the complex anomalies. The final diagnosis was atrial and visceral situs inversus with malposition of the great vessels, DORV, CAVSD with a common atrioventricular (AV) valve, bilateral vena cavae, and PS. She also had pulmonary tuberculosis and a probable congenital scoliosis. This balanced physiology enabled her to survive into adulthood and that any invasive intervention may aggravate her functioning twisted heart. She is on regular follow-up at our clinics for non-invasive monitoring, dental prophylaxis, vaccinations, and continuous counseling for her illness.

## Discussion

### A conundrum of congenital defects

Our patient with multiple congenital defects - visceral and atrial situs inversus with levocardia, malposition of the great arteries, DORV, CAVSD, PS, and bilateral SVC - is unique in that despite its complexity, a balanced circulation has allowed her to survive into early adulthood albeit with exertional cyanosis and dyspnea. To our knowledge, the occurrence of this combination has not been reported in published literature. Data regarding similar lesions are culled from case reports in pediatrics. One is a case, published by Goyal et al [3], of a 16-year-old female who consulted for cyanosis and was subsequently diagnosed with situs inversus with congenitally corrected transposition of the great arteries, dextrocardia, VSD, and PS. Another rare case of a triply reversed heart is a publication of Chang et al [4] who described a 57-year-old acyanotic female with dyspnea. Workup revealed situs inversus with congenitally corrected transposition of the great arteries. Our case is unique in that DORV and malposition of the great arteries are present, making diagnosis and hemodynamics more complicated. Despite the complexity, our patient is alive and functional due to the interplay of the lesions, as individually discussed below.

#### Situs inversus with levocardia

Situs describes the position of the viscera and atria. Situs solitus is the normal position, and situs inversus is the mirror image wherein the morphologic right atrium (RA), inferior vena cava (IVC), and liver are on the left, while the morphologic left atrium (LA) and spleen are on the right [5]. Situs inversus occurs more commonly with dextrocardia, and situs inversus with levocardia is rare (our patient) [6]. In terms of physiology, the presence of isolated situs inversus may be of little clinical significance. Its presence may suggest development of other congenital defects and warrants further imaging.

#### DORV and malposition of the great arteries

DORV is a rare type of ventriculoarterial connection (1-3% of congenital heart defects) wherein both the aorta and pulmonary artery arise predominantly from the RV. In this case, the only outlet of the LV is a septal defect (usually a VSD). The great arteries in DORV may take different relationship. In less than 30% of the time, the aorta is anterior and to the right of the pulmonary artery, as in our case (i.e. malposition of the great arteries) [7].

#### CAVSD

CAVSDs are rare complex cardiac malformations characterized by a deficiency of the atrioventricular septum, accounting for less than 3% of all congenital defects (most of which have the Down syndrome). Patients are symptomatic early in life. Exercise intolerance and congestive heart failure usually start during adolescence [8]. A retrospective report by Sulafa [9] identified 41 patients with AVSD associated with other defects: TOF in 68%, situs inversus in 4%, PS, and DORV. In our case, the natural course is influenced by a CAVSD. Due to the complex nature of her defects, the CAVSD is a double-edged sword. It is a necessary defect to allow proper blood flow which may have allowed our patient to survive up to the present time. On the other hand, it will eventually promote pulmonary hypertension, eisenmengerization, and RV dysfunction.

### Hemodynamics and management of our patient

As mentioned previously, the CAVSD in our case is a necessary defect which may have been the reason why our patient survived into adulthood. This defect created a “functional” single atrium and a “functional univentricle”, leading to a balanced circulation, allowing deoxygenated and oxygenated blood to enter, mix, and leave the heart (Fig. 8 explains the hemodynamics). Anatomically, however, both atria are still separated by a small segment of the interatrial septum. Likewise, both ventricles are also divided by a small segment of the interventricular septum. Despite this small segment dividing the chambers, the atria function as a single atrium and the ventricles function as a single ventricle. The single atrium is connected to the single ventricle via a common AV valve. Fortunately, no significant regurgitation was noted in this single (common valve), which may cause significant hemodynamic consequences. After discussion with a multidisciplinary team, surgery was deemed to put our patient in a high risk for perioperative mortality, taking into account other medical comorbidities such as extensive tuberculosis, malnutrition, and severe scoliosis. Options were presented to the patient and a palliative approach was favored.

### Conclusion

We presented a case of a young adult with cyanosis with a combination of complex congenital heart defects. Diagnosis of our case by echocardiography was a challenge due to the complex nature of the defects, emphasizing that a meticulous and properly done 2D echocardiogram is pivotal to the diagnosis of complex heart diseases. To our knowledge, this is the first reported case in an adult with this combination of anomalies. A palliative approach was favored in the management of this balanced, functioning, twisted heart. The severe PS was deemed protective from Eisenmengerization. Despite the complexity of the defects, she had a balanced circulation which made survival into adulthood possible, albeit with limited functional capacity.
